# PROTACs in Antivirals: Current Advancements and Future Perspectives

**DOI:** 10.3390/molecules30163402

**Published:** 2025-08-18

**Authors:** Jiacheng Jin, Mengxiang Quan, Xueyan Cao, Yun Zhang, Xiangwei Xu, Zunyuan Wang

**Affiliations:** 1 Affiliated Yongkang First People’s Hospital and School of Pharmacy, Hangzhou Medical College, Hangzhou 310013, China; 2 Yongkang First People’s Hospital, Yongkang 321306, China

**Keywords:** PROTAC, targeted protein degradation, antiviral therapy, drug resistance, host–pathogen interaction

## Abstract

Proteolysis-targeting chimera (PROTAC) technology has demonstrated remarkable progress in tumor therapy, attributed to its unique capability of catalytically degrading “undruggable” targets. In the context of the ongoing global health threat posed by the Coronavirus Disease 2019 (COVID-19) pandemic, the application scope of PROTAC technology has been gradually extended to the field of antiviral research. Unlike traditional small molecule inhibitors, PROTAC employs an “event-driven” mechanism to achieve ubiquitination-mediated degradation of target proteins. This approach holds great promise in addressing challenges such as drug resistance, targeting host-dependent factors, and high-mutagenic viral proteins. This article provides a comprehensive review of the application progress of PROTAC technology in antiviral therapy, with a particular emphasis on successful cases across a range of viral pathogens, including Hepatitis B Virus (HBV), Hepatitis C Virus (HCV), influenza virus, and Severe Acute Respiratory Syndrome Coronavirus 2 (SARS-CoV-2). Additionally, it delves into the challenges encountered in this field and ponders future development directions. Through the integration of the latest research findings, this article proposes a dual-target degradation strategy based on the host–pathogen interaction interface. These proposals aim to offer theoretical support for the clinical translation of antiviral PROTACs.

## 1. Introduction

Since the dawn of the 21st century, coronaviruses (CoV) have become notorious for inciting severe pneumonia outbreaks in humans, which have often been fatal [1]. As of 30 April 2025, COVID-19, caused by the “Severe Acute Respiratory Syndrome Coronavirus 2” (SARS-CoV-2), had resulted in over 777 million confirmed cases and seven million deaths worldwide [2]. SARS-CoV, SARS-CoV-2, and Middle East Respiratory Syndrome Coronavirus (MERS-CoV) are categorized as zoonotic, underscoring their animal origins. Vaccination has been the cornerstone of prevention against viral dissemination. Yet, with most vaccines focusing on the viral spike protein (S protein), the advent of the SARS-CoV-2 Omicron variant, characterized by up to 32 mutations in the S protein’s neutralizing antibody target sites, has jeopardized the effectiveness of current vaccines against COVID-19 [3]. This development underscores an ongoing, critical need for the discovery of novel and effective therapeutics.

The pharmacological arsenal against COVID-19 spans a diverse array of strategies, ranging from small-molecule drugs and interferons to vaccines, oligonucleotides, peptides, and monoclonal antibodies [2]. Notably, small-molecule drugs have secured a leading role in contemporary drug development. Their appeal lies in their advantageous properties, including facile absorption, compact size, propensity for cell membrane permeation, and suitability for large-scale industrial synthesis [4].

**Figure 1:**
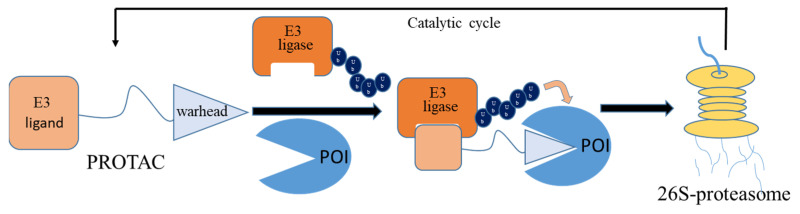
Basic structure of PROTAC and its mechanism of action [6].

Conventional small-molecule inhibitors rely on continuous target engagement to exert therapeutic effects, which can lead to off-target toxicity and resistance. This enduring interaction is often associated with a cascade of side effects and can accelerate the emergence of drug resistance, significantly limiting the therapeutic window of these agents [5]. In contrast, the targeted protein degradation (TPD) strategy has piqued the interest of the scientific community, marking a notable paradigm shift in drug development. Proteolysis-targeting chimeras (PROTACs) have risen to prominence as a vanguard in this therapeutic revolution [6]. PROTACs are composed of three key components: a ligand that binds the target protein, a connector linking this ligand to an E3 ubiquitin ligase, and the E3 ligase ligand itself. Unlike the “occupancy-driven” mechanism of conventional inhibitors, PROTACs operate on an “event-driven” principle, harnessing the ubiquitin–proteasome system (UPS) to orchestrate the degradation of the target proteins (Figure 1). This strategy confers several advantages, including enhanced potency, selectivity, catalytic efficiency, and the capacity to tackle previously “undruggable” targets [6,7]. The allure of this technology has spurred leading pharmaceutical entities to explore its potential, with a burgeoning roster of numerous PROTAC-based therapeutics advancing into clinical trials [8]. These drugs target a spectrum of proteins, such as AR, BCL-XL, IRAK4, STAT3, BTK, TRK, and BRD9. Among them, the most advanced is Vepdegestrant (ARV-471), an ER-targeting degrader developed by Arvinas Ltd. Co. (New Haven, CT, USA), which submitted a New Drug Application (NDA) to the U.S. FDA in June 2025 [9].

The human body encodes over 600 types of E3 ligases. Currently, around 10 published E3 ligases are used for protein degradation research. Protein degraders in the clinical stage mainly target the celeblon (CRBN) and von Hippel–Lindau (VHL) E3 ligases. The application of PROTAC technology in oncology and related diseases has been extensively validated, and its potential in the context of antiviral therapy has attracted substantial attention from academia and industry [10]. Since the first report of using PROTACs for the degradation of the HBV X protein in 2014, this technology has rapidly expanded in the field of antiviral research, particularly demonstrating unique advantages in addressing emerging pathogens such as SARS-CoV-2. This review aims to summarize the current status of PROTAC technology in antiviral research and to propose innovative solutions based on existing technological bottlenecks, with a particular emphasis on successful cases across a range of viral pathogens, including Hepatitis B Virus (HBV), Hepatitis C Virus (HCV), influenza virus, and SARS-CoV-2. Additionally, it delves into the challenges encountered in this field and ponders future development directions. Through integration of the latest research findings, the article proposes a dual-target degradation strategy based on the host–pathogen interaction interface. These proposals are aimed at offering theoretical support for the clinical translation of antiviral PROTACs.

## 2. PROTAC Degrader for Hepatitis Virus

### 2.1. Hepatitis B Virus (HBV)

**Figure 2:**
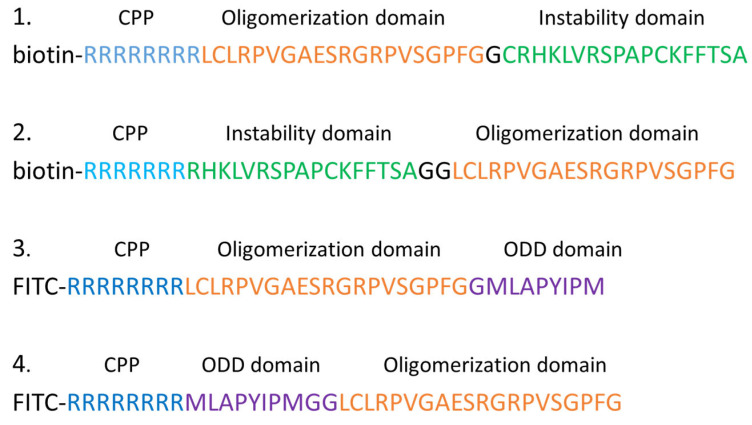
PROTACs based on the instability domain of the X protein (Compd. **1** and **2**), and PROTACs based on the ODD domain of HIF-1a (Compd. **3** and **4**) [11].

In 2014, Montrose et al. [11] first reported the application of PROTAC technology for targeting viral proteins. Given the significant global health burden of hepatocellular carcinoma (HCC) induced by HBV, which results in over one million deaths annually, they developed peptide-based PROTACs to induce the degradation of the HBV-encoded X protein in hepatocytes. The X protein is a crucial target involved in HBV replication and contributes to the progression of liver disease induced by HBV. The researchers constructed four peptide-based PROTACs by fusing the N-terminal oligomerization and C-terminal instability domains of the X protein and conjugated them with polyarginine cell-penetrating peptides (CPPs) to enhance cellular permeability (Figure 2, Compd. **1**–**4**). The design anticipated that the N-terminal oligomerization domain would effectively bind to the X protein, while the C-terminal instability domain would target the X protein for proteasomal degradation. Experimental results have demonstrated that the addition of this PROTAC to HepG2 liver cancer cells successfully induced the degradation of the X protein.

However, this study was limited to a preliminary attempt as it targeted the host cell’s X protein without assessing the PROTAC’s ability to inhibit HBV replication or the development of liver disease. Additionally, the peptide-based PROTAC construct may undergo unpredictable degradation in vivo, presenting significant limitations. Nonetheless, this study serves as a valuable reference for subsequent research on the degradation of viral proteins.

### 2.2. Hepatitis C Virus (HCV)

**Figure 3:**
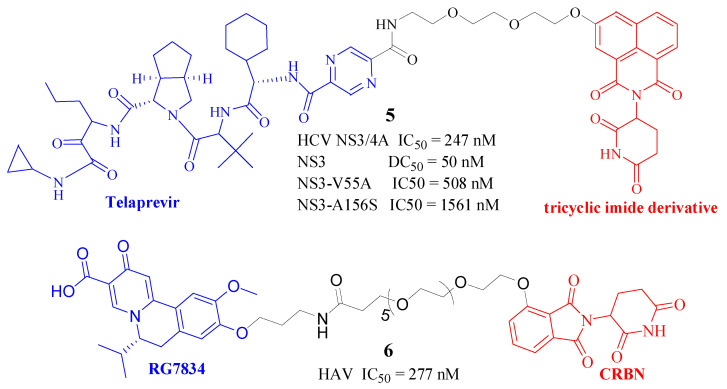
Structure of Compd. **5** and **6**, a potent HCV [12] and HAV [14] degrader, respectively. (Note: Different colors are used to show the three parts of PROTAC, with red for the E3 ligase binding ligand, blue for the target binding ligand, and black for the linker. This is the same in the following figures and will not be repeated. For interpretation of the references to color in this figure legend, the reader is referred to the web version of this article.)

In 2019, a group led by Yang reported the development of the first small-molecule PROTACs with the capacity to degrade viral proteins [12]. Their study targeted the Hepatitis C Virus (HCV) NS3/4A protease, utilizing the small-molecule inhibitor Telaprevir as the binding ligand for the NS3/4A protease. A variety of ligands, including lenalidomide, pomalidomide, and a tricyclic imide derivative, were selected for their ability to bind with the ubiquitin ligase CRBN. This selection facilitated the creation of a series of PROTAC molecules that were effective in targeting and degrading HCV proteins. Significantly, DGY-08-097 (Figure 3, Compd. **5**) emerged as the most potent degrader, exhibiting nanomolar IC50 values against the NS3/4A protease and demonstrating concentration-dependent degradation of the intracellular NS3 protein, a hallmark of the “hook effect” inherent to PROTAC technology.

DGY-08-097 not only preserved its inhibitory activity against the viral protease (IC50: 247 nM) but also induced its degradation via the UPS pathway (DC50: 50 nM). The enhanced activity of this PROTAC was linked to its structural attributes, specifically the use of a tricyclic imide derivative as the CRBN ligand, which provided excellent binding affinity without causing degradation of IMiD substrates. All three PROTAC molecules evaluated exhibited antiviral efficacy at concentrations of 10–40 μM without inducing cellular toxicity. Significantly, these PROTACs also displayed antiviral effectiveness against Telaprevir-resistant HCV strains, underscoring the potential of protein degradation strategies in surmounting drug resistance challenges [13].

### 2.3. Hepatitis A Virus (HAV)

Hepatitis A Virus (HAV) and Hepatitis B Virus (HBV) both affect the liver, yet they exhibit significant differences in their characteristics. HBV is a hepadnavirus, which possesses a DNA genome and necessitates nuclear involvement for replication. Conversely, HAV is a picornavirus with an RNA genome, completing its replication exclusively in the cytoplasm [14]. Dihydroquinolizinones (DHQs), such as RG7834, targeting cellular polyadenylating polymerases 5 and 7, have demonstrated efficacy against both HAV and HBV under experimental conditions, encompassing both in vitro and in vivo studies. Li et al. [14] designed and synthesized RG7834-derived PROTACs, with compound **12b** (Figure 3, Compd. **6**) being a prime example. This innovative DHQ-based PROTAC exhibited potent in vitro inhibition, achieving an IC50 of 277 nM. Although the precursor molecule RG7834 also suppresses HBV, the PROTAC’s in vitro potency was comparatively reduced, with an activity of 16 μM as indicated by diminished levels of hepatitis B surface antigen (HBsAg) and HBV mRNA.

## 3. PROTAC Degrader for Human Cytomegalovirus (HCMV)

**Figure 4:**
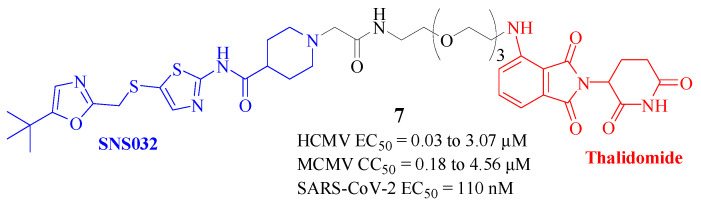
Structure of Compd. **7** for HCMV [15] and SARS-CoV-2 [16] degradation.

Human Cytomegalovirus (HCMV), a ubiquitous herpesvirus, is associated with a range of clinical manifestations. The existing antiviral treatments have frequently been found to be inadequate in addressing the full scope of medical requirements. In 2021, Professor Manfred Marschall and colleagues at the University of Erlangen-Nuremberg, Germany, reported a novel strategy utilizing PROTAC technology to target HCMV [15]. This approach was centered on the CDK inhibitor SNS032 for the degradation of HCMV proteins. The molecule that displayed the most promising results was THAL-SNS032 (Figure 4, Compd. **7**), which showed potent anti-HCMV activity, marking a 3.7-fold improvement in efficacy over the non-PROTAC molecule SNS032. THAL-SNS032 selectively degraded CDK9 at lower concentrations, while still retaining broad-spectrum inhibitory activity against CDK2/7/9. Additionally, this PROTAC exhibited antiviral activity against other viruses, including the inhibition of SARS-CoV-2 replication, without affecting human viruses such as VZV, HAdV-2, and ZIKV. THAL-SNS032 also demonstrated a more stable and enduring efficacy profile when compared to SNS032, a conventional CDK inhibitor [16]. However, the swift therapeutic onset of SNS032 suggested that further research is necessary to fully understand the pharmacokinetics and pharmacodynamics of THAL-SNS032.

## 4. PROTAC Degrader for Influenza Virus

Influenza, commonly known as the flu, is an acute respiratory infection caused by the influenza virus. It represents a substantial threat to the health of both humans and animals, with significant impacts on poultry, pigs, and chickens [17]. The influenza virus is a member of the Orthomyxoviridae family and is characterized as a negative-sense, enveloped RNA virus. It is further classified into 18 hemagglutinin (HA) and 11 neuraminidase (NA) subtypes based on the antigenic properties of its surface glycoproteins. To date, the FDA has approved a limited number of antiviral medications for influenza treatment, which includes two M2 proton channel inhibitors (amantadine and rimantadine), three neuraminidase inhibitors (zanamivir, oseltamivir, and peramivir), and one RNA-dependent RNA polymerase inhibitor (baloxavir marboxil). However, the obsolescence of M2 inhibitors due to severe side effects and the escalating resistance to neuraminidase inhibitors underscore the urgent requirement for the development of novel anti-influenza agents that have both low toxicity and high potency [17].

### 4.1. PROTAC Degraders for the Influenza Virus NA

**Figure 5:**
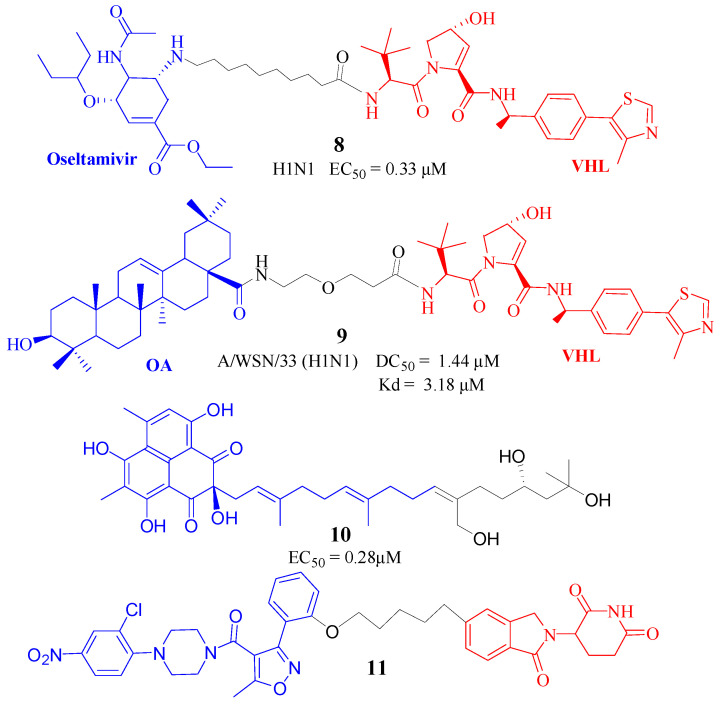
Structure of Compd. **8**–**11** for influenza virus [17,18,19,20].

Researchers led by Zhou at Wuhan University [17] utilized PROTAC technology to synthesize antiviral PROTAC compounds by linking the neuraminidase inhibitor oseltamivir with ligands for the ubiquitin ligases VHL or CRBN. These compounds underwent rigorous testing for cytotoxicity, antiviral efficacy, and protein degradation capacity, with oseltamivir and amantadine serving as positive controls. The findings revealed that the majority of the compounds displayed remarkable inhibitory effects against the H5N1 subtype of the influenza virus, outperforming the standard treatments of oseltamivir and amantadine. Importantly, significant inhibitory effects were also observed against strains resistant to oseltamivir (H5N1 H274Y) and amantadine (PR8-H1N1). In vitro studies demonstrated the degradation of the NA protein, with most compounds showing low DC50 values (below 2.27 μM), which positions them as potential degraders of the influenza virus NA. Notably, compound 8e (Figure 5, Compd. **8**) demonstrated the most potent activity with an EC50 of 0.33 μM and minimal toxicity to normal cells, suggesting its promising candidacy for the development of new influenza antiviral pharmaceuticals.

### 4.2. PROTAC Degraders for the Influenza HA

The influenza HA, a key facilitator of viral entry into host cells via membrane fusion, has emerged as a promising target for antiviral therapeutics. In 2022, Li et al. [18] investigated the potential of PROTAC technology to degrade the HA protein associated with the influenza virus. They employed a strategic approach by linking oleanolic acid (OA), a natural product known to inhibit viral entry, with E3 ubiquitin ligases CRBN and VHL. This innovative method enabled the targeted intracellular degradation of the hemagglutinin protein through the action of PROTACs, effectively extending the antiviral efficacy of oleanolic acid from the extracellular environment into the cell. The resulting PROTAC molecules maintained their capacity to bind both the hemagglutinin target proteins and E3 ligases, facilitating the degradation of hemagglutinin at the molecular and cellular levels. Notably, compound V3 (Figure 5, Compd. **9**) demonstrated potent degradation of the A/WSN/33 (H1N1) virus protein during the replication phase, with a DC50 of 1.44 μM, a dissociation constant (kD) of 3.18 μM, and a half-life (T1/2) of 2.87 h. Through a series of experiments, including immunoprecipitation, proteasome inhibition, and VHL enzyme knockout, the study confirmed that the degradation of the hemagglutinin protein by these PROTAC molecules was dependent on the UPS. Furthermore, intravenous administration of V3 was shown to protect mice from the lethal effects of the influenza A virus. Pharmacokinetic and pharmacodynamic studies revealed that these molecules possess oral bioavailability, exhibit broad-spectrum activity against the influenza A virus, and provide protective effects in mouse weight and survival rates by reducing viral loads and lung inflammation. Collectively, these findings suggest that PROTACs based on oleanolic acid hold potential as a novel direction for the development of anti-influenza drugs targeting the HA protein.

In 2022, compound APL-16-5 (Figure 5, Compd. **10**) was identified from *Aspergillus* sp. CPCC 400735 to possess antiviral properties against influenza A virus (IAV) with an EC50 of 0.28μM [19]. This activity is mediated by its interaction with the E3 ubiquitin ligase TRIM25, which facilitates the ubiquitin-dependent degradation of the IAV polymerase. Remarkably, APL-16-5 also protected infected mice with a survival rate of 100% at a dose of 20 or 100 mg/kg and reduced the severity of pulmonary inflammation. Although APL-16-5 represents a nontraditional example of a rationally designed PROTAC, it validated the potential of TPD as a therapeutic strategy against influenza viruses, and microbial metabolites could be an important source for the discovery of natural PROTAC molecules.

Nucleozin, recognized for its effectiveness against IAV, acts by targeting the nucleoprotein (NP) and inducing its aggregation. In a strategic approach, Zhao et al. [20] utilized Nucleozin as a molecular handle to craft PROTACs. They developed a series of Nucleozin- and CRBN-based PROTACs. A novel small molecule, FM-74-103 (Figure 5, Compd. **11**), emerged as a selective degrader of GSPT1, a factor pivotal in the transition from G1 to S phase of the cell cycle, and demonstrated inhibitory capabilities against a spectrum of RNA and DNA viruses. Notably, FM-74-103 maintained its anti-IAV potency against a drug-resistant strain, A/Netherlands/602/2009 (H1N1), characterized by a mutation in the NP (Y289H), which confers resistance to Nucleozin. The broad-spectrum antiviral profile of FM-74-103 extends to SARS-CoV-2, where it was observed to curtail viral replication in A549-ACE2 cells with minimal cytotoxic effects. The mechanism of action involves GSPT1 degradation and the modulation of eIF2α, a key component in the cellular response to viral infection. Additionally, compound FM-74-103 has shown activity against Cytomegalovirus (CMV). Collectively, by orchestrating the depletion of GSPT1, compound 103 presents a robust defense against a variety of viral infections in human cells.

Beyond the employment of small molecules to target viral proteins for the development of PROTAC-based antiviral strategies, a novel approach was further introduced by integrating RNA oligonucleotides into the PROTAC framework [20]. This innovative design leverages the untranslated regions (UTRs) of IAV RNA as a starting point. The IAV UTRs, both 5′ and 3′, serve as promoters that attract the viral polymerase (vPOL), which is essential for the transcription and replication of viral RNA (vRNA). The rationale for this design stems from the observation that the UTRs of IAV are highly conserved across different segments of the virus and exhibit relatively slow rates of evolution compared to the coding regions. Moreover, these sequences maintain a high level of conservation across various viral strains and host species. Due to their critical role in vPOL recruitment and their exceptional conservation, these UTR sequences have been identified as superior ligands for the creation of targeted antiviral molecules.

### 4.3. PROTAC Viral Vaccine

Despite advancements in influenza vaccine development and their widespread administration, influenza viruses continue to pose a substantial threat to global public health. Conventional prevention strategies, such as immunization with inactivated influenza vaccines (IIVs) and live attenuated influenza vaccines (LAIVs), remain cornerstones in controlling seasonal outbreaks. However, these methods face limitations in offering protection against emerging strains of influenza with pandemic potential, largely due to the high variability of the virus [21]. In 2022, Si et al. [22] explored the innovative use of PROTAC technology to engineer a live attenuated A-type influenza virus vaccine by targeting viral protein degradation. They introduced a proteasome-targeting domain (PTD) into the M1 protein gene segment of the IAV, designed to be recognized by the E3 ubiquitin ligase VHL, thereby promoting the ubiquitination and subsequent degradation of IAV proteins associated with the M1 gene segment. This novel construct was termed a PROTAC vaccine.

**Figure 6:**
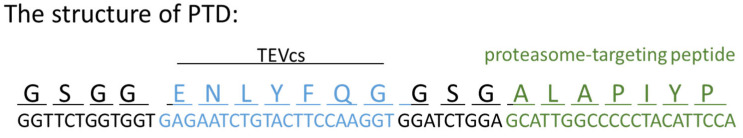
The structure of the PTD part of Compd. **12** [22].

To facilitate vaccine production, a tobacco etch virus (TEV) cleavage site was engineered onto the PTD, allowing for selective excision by the TEV protease (TEVp) and preserving the virus’s capacity for efficient replication in MDCK cells stably expressing TEVp. Employing this design rationale, the research team initially developed a strain of the PROTAC influenza virus vaccine, designated M1-PTD (Figure 6, Compd. **12**). Analysis of the viral growth curve confirmed that M1-PTD was capable of efficient replication exclusively in cells optimized for PROTAC virus propagation, with markedly diminished replication in standard cells, thereby ensuring safety. Immunofluorescence assays further revealed the degradation of M1-PTD viral proteins within normal cells. Plaque assays indicated that M1-PTD was restricted to forming plaques in cells designed for PROTAC virus propagation, with no plaque formation in normal cells. Additionally, cell viability assays demonstrated that M1-PTD did not exert significant cytopathic effects on normal cells. Collectively, these findings suggest that the M1-PTD influenza virus holds promise as a potential safe vaccine candidate. Animal safety assessments, including mouse and ferret models, demonstrated that this PROTAC-based viral vaccine technology effectively attenuated the IAV, resulting in minimal viral replication without inducing death or weight loss in the animal models, thereby showcasing its favorable safety profile.

This study, grounded in the concepts of synthetic biology, paved the way for a new approach to vaccine development utilizing protein degradation-targeted viruses as a means to create live attenuated vaccines, using the influenza virus as a model. However, it is important to note that the use of the PTD sequence, which targets VHL, could limit the applicability of this PROTAC vaccine in certain individuals, such as those receiving treatment with proteasome inhibitors or those with specific E3 ligase expression abnormalities [23].

## 5. PROTAC Degrader for SARS-CoV-2

In response to the persistent challenges of the highly mutable and exceptionally contagious SARS-CoV-2 virus, researchers are investigating the potential of pioneering PROTAC technology as a combative strategy against infection. Current research endeavors are primarily divided into two strategic avenues: targeting the degradation of host cell proteins and targeting the degradation of viral proteins.

### 5.1. PROTACs Targeting the Degradation of Host Proteins

#### 5.1.1. PROTACs Targeting ACE2

Research into the mechanisms of viral invasion has revealed that coronaviruses predominantly utilize their spike protein (S protein) to engage with the angiotensin-converting enzyme 2 (ACE2) receptor on host cells, facilitating viral entry. Soluble ACE2 protein (sACE2) has been recognized as a competitive inhibitor for SARS-CoV-2 and other coronaviruses, preventing the virus from binding to endogenous ACE2 transmembrane proteins and thereby inhibiting viral entry into host cells [24]. Chatterjee et al. [25] employed computational protein modeling to devise minimal ACE2 peptides capable of simultaneously targeting the virus spike protein receptor-binding domain (RBD) and recruiting E3 ubiquitin ligases. This strategy induced the degradation of SARS-CoV-2 proteins within the host cell’s proteasome. An optimal peptide variant, comprising 23 amino acids (sequence structure: QAKTFLDKNHEADLFYQSSLA), was identified, which significantly enhanced the degradation of the RBD fused with stable superfolder green fluorescent protein (sfGFP) in human cells, thereby suppressing the generation of infectious viruses. Although this peptide fusion platform serves as a preventive strategy, it is important to note that it employs the UPS akin to PROTAC methodology, yet it lacks the characteristic “three-part” molecular architecture of traditional PROTAC molecules.

#### 5.1.2. PROTAC Targeting TMPRSS2

In the process of CoV invasion, the transmembrane protease serine 2 (TMPRSS2) protein in host cells played a key role in activation. Blocking the efficiency of the TMPRSS2 protein has seemed an effective way to treat SARS-CoV-2 and a universal method to resist virus infection in the human body [26]. However, the current small-molecule inhibitors, such as Camostat, Nafamostat, and Bromohexin Hydrochloride (BHH), were still not strong enough to inhibit the activity of TMPRSS2, even though it is easy to induce secondary drug resistance [27].

**Figure 7:**
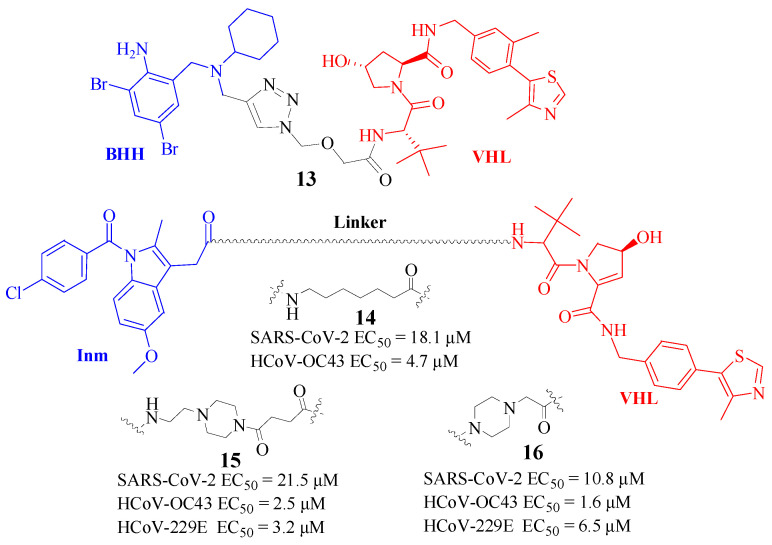
Structure of Compd. **13**–**16** as host-directed PROTACs degraders.

Zhang et al. [28] licensed a Chinese patent to modify BHH, which was both a widely used expectorant drug and an efficient TMPRSS2 inhibitor, into a PROTAC. They first introduced the propyne group on BHH and then connected it to the VHL ligand by click reaction to obtain the desired PROTAC compound. Protein degradation experiments showed that the target compound d1 (Figure 7, Compd. **13**) could show significant TMPRSS2 protein degradation at 0.1 μM concentration in experiments with the administration of different concentrations. In an experiment on different administration times, the target compound showed significant TMPRSS2 degradation at 4 h.

#### 5.1.3. PROTAC Targeting PGES-2

Indomethacin (Inm, also known as Indocin), a nonsteroidal anti-inflammatory drug, has been implicated in the inhibition of the early stages of the coronavirus replication cycle with weak SARS-CoV-2 inhibitory activity (EC50 ∼ 100 μM). This effect was proposed to be due to Inm’s ability to inhibit human prostaglandin E synthase-2 (PGES-2), which interacted with the NSP7 protein of the SARS-CoV-2 virus [29]. Capitalizing on these insights, Shaheer et al. [30] synthesized the inaugural series of PROTAC-based molecules targeting SARS-CoV-2. By conjugating Inm with the E3 ubiquitin ligase VHL through alkyl and PEGylation linker chains, they developed four PROTAC molecules and assessed their anti-SARS-CoV-2 activity. Compound 3 (featuring a 6-methylene linker unit, Figure 7, Compd. **14**) and compound 5 (with a piperazine-based linker, Figure 7, Compd. **15**) demonstrated approximately 4.5-fold greater potency than Inm. Furthermore, these compounds exhibited broad-spectrum inhibitory effects against other human CoVs, including β-coronavirus HCoV-OC43 (EC50 of 4.7 μM and 2.5 μM, respectively) and α-coronavirus HCoV-229E (EC50 of 36.5 μM and 3.4 μM, respectively). This suggests that compounds 3 and 5 possess broad-spectrum antiviral properties against both pandemic and endemic CoVs from different genera within the Coronaviridae family.

Recently, a further study [31] on Inm–based PROTACs was reported by the same group. Their study expanded the repertoire of Inm-based PROTACs by exploring various E3 ligands and linker moieties. PROTAC 6 (Figure 7, Compd. **16**), similar to its predecessors PROTACs 3 and 5 (Figure 7, Compd. **14** and **15**, respectively), demonstrated broad-spectrum antiviral efficacy against CoVs, including SARS-CoV-2 with an EC50 of 10.8 μM, and two distinct endemic human CoVs, HCoV-OC43, and HCoV-229E, with EC50 values of 1.6 μM and 6.5 μM, respectively. Importantly, these effects were observed at concentrations that were not toxic to cells. The antiviral potency of PROTAC 6 against SARS-CoV-2 was further validated in a human cell line, with an EC50 in the nanomolar range, specifically 890 nM. In contrast, no active degraders were produced when Inm was conjugated with a CRBN recruiter.

However, Western blot analysis on both uninfected and SARS-CoV-2-infected cells revealed that these Inm–based PROTACs did not degrade the initially suspected human pPGES-2 protein. Instead, they induced concentration-dependent degradation of the SARS-CoV-2 main protease (Mpro), as evidenced in both Mpro-transfected cells and those infected with SARS-CoV-2.

### 5.2. PROTAC Targeting the Degradation of Viral Proteins

#### 5.2.1. PROTAC Targeting Mpro

The SARS-CoV-2 virus, responsible for the COVID-19 pandemic, is composed of four main structural proteins: the spike protein (S), envelope protein (E), membrane protein (M), and nucleocapsid protein (N). A pivotal enzyme in the viral life cycle is Mpro, also known as 3C-like protease (3CLpro), which is crucial for viral polyprotein processing. This enzyme initially autocatalyzes its release from the polyprotein and subsequently cleaves at over 11 sites, generating essential replication and transcription proteins. Given its minimal homology with human proteins, Mpro is a prime target for anti-coronavirus drug development [32].

In a 2020 review by Liu et al. [33], PROTAC degraders were proposed as a next-generation strategy for anti-CoV therapies. The review mentioned a reversible covalent PROTAC designed for Mpro in their laboratory. However, the structure and activity were not disclosed, and no subsequent reports have been identified.

In 2021, Shaheer et al. [34] utilized computer-aided drug design (CADD) methods to lay the groundwork for designing PROTACs that target Mpro. They employed protein–protein docking to predict the compatibility between the cereblon E3 ligase and SARS-CoV-2’s Mpro, estimating potential linker lengths. Molecular dynamics simulations of the resulting ternary complexes showed stable interactions, suggesting the feasibility of the designed PROTACs to induce target protein degradation. While this computational study has yet to be experimentally verified and the computational strategies for PROTAC design remain in their infancy, it underscores the potential of PROTAC technology in targeting protein degradation pathways for SARS-CoV-2.

**Figure 8:**
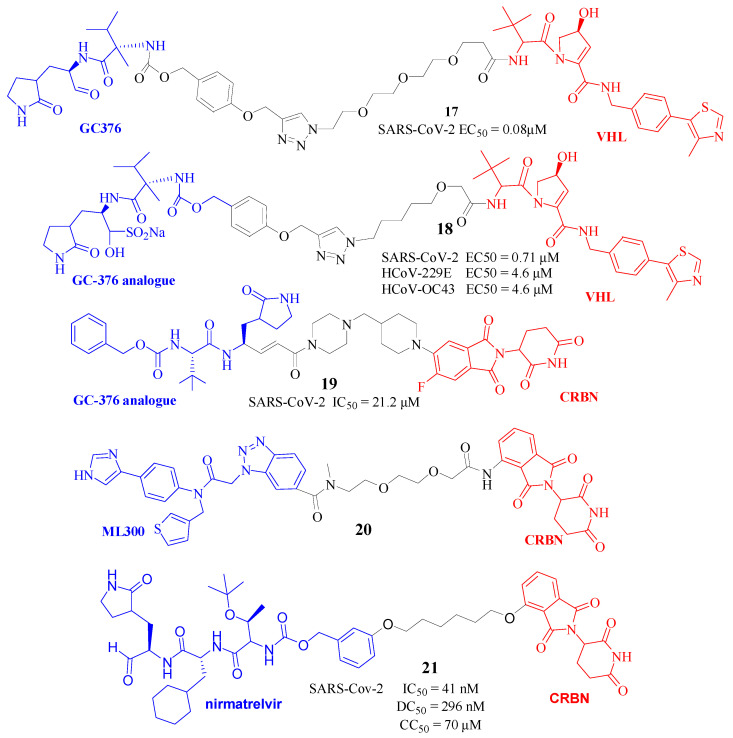
Structures of Compd. **17**–**21** as virus-directed PROTACs.

Since there are active residues, such as Cys145 in the catalytic domain of the Mpro, an effective strategy for developing MPro inhibitors involves the formation of a covalent adduct [35]. GC376, a covalent inhibitor of SARS-CoV-2 Mpro, has demonstrated the ability to inhibit viral replication in vitro [36]. However, GC376 faces challenges in completely blocking viral replication and may not fully suppress the viral infection process. Additionally, structural modifications to GC376 often fail to achieve complete inhibition of viral replication. These modifications may impede GC376’s access to the active site of Mpro, disrupt its interaction with the active site, and/or alter its mechanism of action, resulting in diminished binding affinity and ineffective inhibition of Mpro. To overcome these challenges, Li et al. [37] employed computational chemistry to thoroughly investigate the interaction between GC376 and SARS-CoV-2’s Mpro protease. They designed and synthesized a series of PROTAC molecules, leveraging VHL ligands, and connected them to specific sites on GC376 and its derivatives through various nature-specific linkers to the E3 ubiquitin ligase. Cellular experiments indicated that most of these PROTAC molecules effectively inhibited the activity of the novel coronavirus, with some compounds showing even stronger inhibitory activity than GC376 in vitro. Notably, compound B1-C9-V (Figure 8, Compd. **17**) exhibited the most potent activity with an EC50 of 0.08 μM. Due to its distinct mechanism of action compared to GC376, this new class of PROTAC compounds offered an alternative effective strategy to impede the replication and infection of variant viruses, including emerging strains of the novel coronavirus.

By modifying the inhibitor GC376 at a non-interacting site to create PROTACs, Cheng et al. [38] developed a series of Mpro PROTACs with relatively broad-spectrum effectiveness against HCoVs, including HCoV-229E, HCoV-OC43, and SARS-CoV-2. The EC50 of this PROTAC (Figure 8, Compd. **18**) ranged from 0.71 to 4.6 μM, and neither showed cytotoxicity at 100 μM. Furthermore, degrading mechanism studies demonstrated that these PROTACs effectively reduced Mpro degradation within cells in vitro.

Further PROTAC molecule was designed (Figure 8, Compd. **19**) by conjugating a GC-376-based dipeptidyl 3CLpro ligand to a CRBN moiety through a piperazine–piperidine linker [39]. A docking study showed that the linker and the pomalidomide cereblon-ligand component of the PROTAC molecule extended outwards from the protein, exhibiting a significant level of flexibility without forming interactions with other proteins. Additionally, this PROTAC effectively diminished the protein levels of the SARS-CoV-2 3CLpro enzyme in cultured cells. These findings are instrumental in establishing the potential for peptidomimetic PROTACs as a therapeutic strategy against 3CLpro-dependent viral infections, thereby broadening the scope of antiviral treatment options.

Given that the successful degrader design requires a thorough understanding of the target, Khurshid et al. [40] highlighted representative examples of a full TPD design. Based on in silico analysis, they selected the tertiary arylamide inhibitor ML300 derivative as the warhead and Pomalidomide as E3 ligase ligands. Thus, PROTACs with linkers of varying length were generated for further evaluation via computational modeling and the scoring of the resulting ternary complexes. A prospective ML300-based degrader (Figure 8, Compd. **20**) was proposed, and further predictions indicated that this PROTAC had overall favorable properties, including solubility, permeability, first-pass metabolic stability, and clearance. However, the compound was only a putative one. It should actually be synthesized, and the bioactivities verified.

Most recently, utilizing PROTAC technology, Xu’s group [41] engineered a novel series of small-molecule antivirals designed to trigger the degradation of the Mpro. They also utilized an aldehyde group as an electrophilic warhead to form a reversible covalent bond with the active site Cys145 and designed ten Mpro PROTAC degraders: MPD1 to MPD10. Notably, compound MPD2 (Figure 8, Compd. **21**) has been shown to significantly lower MPro levels within 293T cells through a process that is time-dependent and reliant on CRBN-mediated recognition and proteasomal degradation. Additionally, MPD2 has demonstrated significant effectiveness in reducing Mpro protein levels in SARS-CoV-2-infected A549-ACE2 cells. The compound has also proven effective against a spectrum of SARS-CoV-2 strains and has shown increased potency against strains resistant to the antiviral nirmatrelvir. Collectively, this study underscores the promise of Mpro-targeted protein degradation as a pioneering strategy for the development of antivirals capable of combating drug-resistant SARS-CoV-2 variants.

#### 5.2.2. PROTAC Targeting Envelope Protein E

In addition to the Mpro protein, Martinez-Ortiz et al. [42] suggested that the SARS-CoV-2 structural envelope protein E could be a viable target for PROTAC design. Their rationale was multifaceted: Envelope protein E has been effectively targeted in the context of SARS infections. It is unique among the viral envelope proteins for lacking glycosylation, which may facilitate small-molecule binding, and its degradation is hypothesized to disrupt multiple viral processes, including entry, replication, and assembly. However, this concept remains theoretical as they proposed the idea without designing or synthesizing actual PROTAC molecules to test their hypothesis.

## 6. PROTAC Degrader for Flaviviruses

Flaviviruses, including Dengue virus (DENV), Zika virus (ZIKV), Japanese encephalitis virus (JEV), West Nile virus (WNV), and yellow fever virus (YFV), represent a substantial class of human pathogens. Despite their impact, effective preventative and therapeutic measures remain elusive [43]. The genetic and serological diversity of these viruses presents substantial challenges to the development of vaccines and antiviral medications. A key component of the flavivirus life cycle is the envelope (E) protein, which is present in 90 prefusion dimers on the virion’s surface and plays a crucial role in various stages of viral replication.

**Figure 9:**
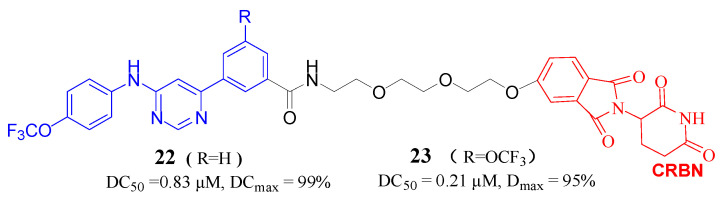
Structures of Compd. **22** and **23** as degraders for Flaviviruses.

A study by Liu et al. [43] detailed the characterization of PROTACs designed to promote the degradation of the DENV E protein by the proteasome. Two of these degrader candidates, referred to as GNF-2-deg and 2-12-2-deg (Figure 9, Compd. **22**, **23**), were found to reduce the intracellular levels of the E protein in a concentration-dependent manner, with this reduction being dependent on the CRBN protein. Notably, both GNF-2-deg and 2-12-2-deg demonstrated a marked enhancement in antiviral efficacy compared to their parent compounds, with a potency increase ranging from 4 to 10 times. Furthermore, the antiviral efficacy of these degraders was not diminished by specific point mutations in the E protein (E-F193, E-M196V, and E-F279S). The research indicated that the E protein degraders significantly outperformed their parent inhibitors in terms of antiviral activity against a spectrum of mosquito-borne flaviviruses, including ZIKV, JEV, WNV, and YFV.

## 7. PROTAC Degrader for HIV

Acquired immune deficiency syndrome (AIDS) is a severe and life-endangering disease. Human immunodeficiency virus-1 (HIV-1), classified as a retrovirus within the lentivirus group, is recognized as the main cause of AIDS [44].

**Figure 10:**
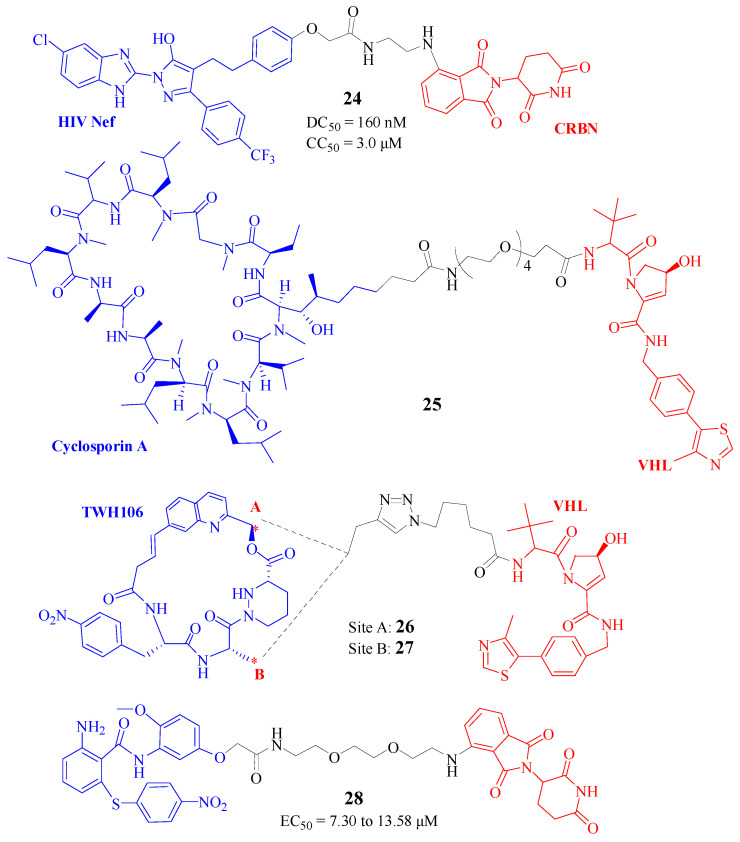
Structures of Compd. **24**–**28** as degraders for HIV. (Note: Compd. **26** and **27** differ only at the connect site A or B, as labelled with an asterisk (*) in the figure.)

The HIV-1 Nef protein, a critical accessory factor, plays an indispensable role in the viral lifecycle, facilitating immune evasion in infected cells. It presents a compelling target for the development of antiretroviral medications. However, because Nef lacks intrinsic enzymatic activity and an active site, it is difficult to discover an inhibitor targeting HIV-1 Nef [45]. To address this, Emert-Sedlak et al. [44] adopted an alternative approach to selectively degrade the Nef protein. A library of 102 PROTACs, based on an existing hydroxypyrazole scaffold that could bind to Nef, and ligands for the CRBN and VHL components of ubiquitin E3 ligases, were designed and screened for the induction of Nef ubiquitylation. In T cell-based assays, these Nef-directed PROTACs efficiently rescued Nef degradation. The most active Nef PROTAC, FC-14369 (Figure 10, Compd. **24**), has shown a DC50 value of 160 nM and a CC50 value of approximately 3.0 μM, indicating that targeted degradation of Nef occurs without cytotoxicity. These PROTACs were also found to enhance the HIV-1 replication in donor PBMCs. In an HIV-1 infectivity assay, FC-14369 could inhibit HIV-1 infectivity with an IC50 value of about 250 nM without cytotoxicity. Importantly, these Nef PROTACs could bind to a diverse panel of HIV-1 Nef variants as well as SIV Nef, indicating they will be broadly active against multiple HIV-1 strains through a shared structure feature within Nef.

Cyclophilin A (CypA), acting as the cellular receptor for the immunosuppressive agent cyclosporin A (CsA), is a plentiful cytosolic protein that plays a role in multiple diseases, including HIV-1 and HCV activity [46]. Given its high abundance within cells, achieving a therapeutic impact necessitates substantial drug concentrations and dosages, which may subsequently result in unwanted side effects. Hilbig et al. [46] designed a series of PROTACs aimed at inducing the intracellular breakdown of CypA. Among these, compound P3 (Figure 10, Compd. **25**) showed the most potent degradation, effectively depleting CypA in lymphocytes without disrupting cell growth or cytokine production. Though this study did not test the antiviral effects, these PROTACs had the potential to serve as influential therapeutic agents.

Newton et al. [47] designed and characterized antiviral PROTACs targeting CypA, CG167 (Figure 10, Compd. **26**), and RJS308 (Figure 10, Compd. **27**), based on TWH106, which is a fully synthetic macrocycle derived from sanglifehrin A. In Jurkat cells, RJS308 treatment almost completely degraded CypA within 24 h, whereas CG167 activity was slower, requiring 4–5 days for a similar degree of CypA degradation. CypA DC50s for the 48 h treatments were 284 nM (RJS308) and 123 nM (CG167) with Dmaxs of 98% (RJS308) and 76% (CG167). Both CG167 and RJS308 degrade CypA via a PROTAC mechanism involving VHL recruitment. Specificity examination showed that CypA was the most PROTAC-sensitive protein detected of the 17 human cyclophilins. These Cyp-PROTACs showed antiviral activity against HIV-1 replication in primary human CD4+ T cells and against HCV replicon in Huh7 cells. This work provides a proof of concept for PROTACs targeting CypA to inhibit infection and will be a useful tool to probe isoform-specific cyclophilin biology [47].

Vif, a viral protein crucial for HIV-1 pathogenesis, has emerged as a promising target for drug development in HIV-1 patients. Luo et al. [48] previously identified a distinctive collection of Vif inhibitors that displayed potent antiviral activity within HIV-1-infected cells. Based on these findings, they designed and synthesized a novel series of Vif-directed PROTACs. Among them, L15 (Figure 10, Compd. **28**) demonstrated a significant concentration-dependent reduction in Vif protein levels, coupled with perceptible antiviral capabilities. L15 has been shown to curb the proliferation of various drug-resistant HIV-1 strains with EC50 values ranging from 7.30 to 13.58 μM, while exhibiting low cytotoxicity, with CC50 values higher than 200 μM. Further mechanistic exploration verified that L15 induced the degradation of Vif via the ubiquitin pathway. It is hypothesized that L15 could provide antiviral benefits across the entire HIV-1 infection process, with an accentuated impact during the initial phases. This study represents a pioneering foray into utilizing a Vif-targeted PROTAC against HIV-1, thus validating the concept of developing antiviral treatments employing the PROTAC methodology for the management of HIV/AIDS.

## 8. Conclusions and Perspectives

In summary, infectious diseases, particularly those caused by coronaviruses, remain a significant threat to global public health. While vaccination campaigns have been largely successful and a number of potent small-molecule drugs are available, the virus’s propensity for mutation and the emergence of resistance call for the development of innovative treatment strategies. TPD technologies, exemplified by PROTAC, are emerging as a potent approach and the next frontier for antiviral therapies, which offer a paradigm shift from “inhibition” to “elimination” in antiviral therapy [40,49]. The studies described above have presented proofs of concept that TPD can be exploited to develop both host-directed and virus-specific strategies against viral infections.

Host-directed PROTACs degrade cellular proteins that viruses exploit for replication. Their greatest advantage is a high genetic barrier to resistance, because host genes mutate far more slowly than viral genomes; a single compound can therefore maintain potency across viral strains and even across unrelated viruses sharing the same dependency. The downside is potential on-target toxicity, as the host protein may perform essential housekeeping functions, and partial loss can elicit adverse events. In addition, pharmacokinetic variability among patients can shift the therapeutic window, complicating dose selection [47]. Virus-directed PROTACs, in contrast, eliminate pathogen-encoded proteins that are absent from human cells. This specificity minimizes intrinsic toxicity and allows aggressive dosing, yet it comes at the cost of a low resistance barrier. Viral polymerases are error-prone, and single-nucleotide substitutions can abolish PROTAC binding or disrupt ternary-complex formation. Consequently, these compounds may require combination regimens or iterative medicinal chemistry to keep pace with emerging variants [19]. Ultimately, the choice between the two strategies will hinge on balancing breadth and safety against durability and mutational risk. Due to the high mutability of viruses, PROTAC technology that targets the degradation of host proteins, thereby blocking the pathways of viral invasion, is more attractive [28]. Designing bispecific PROTACs that simultaneously target both viral proteins and host factors (e.g., SARS-CoV-2 Mpro/ACE2) to reduce the risk of resistance may be an innovative solution.

Across all antiviral PROTACs reported to date, three unifying features stand out: (i) each molecule must nucleate a stable ternary complex with an E3 ligase and the chosen viral or host target [50]; (ii) degradation is strictly ubiquitin- and proteasome-dependent, regardless of the viral species or protein class; and (iii) potency generally tracks with the intrinsic half-life of the substrate—short-lived viral enzymes are degraded more rapidly and at lower concentrations than long-lived host scaffolds [51]. Where the programs diverge is in their selectivity–resistance trade-off [13]. Linker length and E3 ligase choice further differentiate the agents: VHL-recruiting PROTACs tend to favor hydrophobic viral pockets, whereas CRBN-based systems are preferred for host kinases [52]. These convergences and contrasts underscore the need for context-specific optimization and, ultimately, combination strategies that exploit both paradigms.

Nevertheless, the application of PROTAC technology is not without challenges, since PROTACs are a relatively new class of drugs. These challenges include a scarcity of suitable E3 ligand binders, synthetic difficulties, the large molecular size of PROTACs, the potential for off-target toxicity, etc. [10]. Additionally, the development of PROTACs requires a deep understanding of the target protein and its interactions, and the effectiveness of PROTACs requires cell membrane permeability to facilitate intracellular protein degradation, yet increased molecular weight can lead to reduced water solubility and cellular uptake, resulting in diminished bioavailability. Also, their safety and efficacy in humans are still being evaluated. Until now, no PROTAC-based antiviral therapies have been approved for clinical use [53].

Recent advancements in PROTAC technology, such as AutoTAC, CLIPTAC, and nucleic acid-based PROTACs, hold promise for overcoming these limitations and broadening the applicability of PROTACs [54]. Although TPD at the host–pathogen interface is in a nascent stage compared to its more established role in oncology, the continued growth and evolution of PROTAC technology in antiviral research are anticipated.

In conclusion, PROTAC technology, with its unique mechanism, shows great promise in the field of antiviral therapy. Despite the challenges in molecular optimization and clinical translation, innovative design and interdisciplinary technological integration make PROTACs a potential next-generation core therapy for combating viral mutations and resistance. Further exploration of their global regulatory potential in host–virus interaction networks will drive antiviral treatment into an era of precise degradation. Newer TPD techniques are expected to open up additional avenues for the development of antiviral drugs.

## Data Availability

No new data were created or analyzed in this study. Data sharing is not applicable to this article.

## Abbreviations

The following abbreviations are used in this manuscript:

3CLpro	3C-like protease;
ACE2	Angiotensin-converting enzyme 2;
AIDS	Acquired immune deficiency syndrome;
BHH	Bromohexin Hydrochloride;
CADD	Computer-aided drug design;
CMV	Cytomegalovirus;
CoV	Coronaviruses;
COVID-19	Coronavirus Disease 2019;
CPPs	Cell-penetrating peptides;
CRBN	Celeblon;
CsA	Cyclosporin A;
CypA	Cyclophilin A;
DENV	Dengue virus;
DHQs	Dihydroquinolizinones;
E	Envelope protein;
HA	Hemagglutinin;
HAV	Hepatitis A virus;
HBV	Hepatitis B Virus;
HCC	Hepatocellular carcinoma;
HCMV	Human Cytomegalovirus;
HCV	Hepatitis C Virus;
HIV-1	Human immunodeficiency virus-1;
IAV	Influenza A virus;
IIV	Influenza vaccines;
JEV	Japanese encephalitis virus;
LAIV	Live attenuated influenza vaccines;
M	Membrane protein;
MERS-CoV	Middle East Respiratory Syndrome Coronavirus;
Mpro	Main protease;
N	Nucleocapsid protein;
NA	Neuraminidase;
NDA	New Drug Application
OA	Oleanolic acid;
PGES-2	Prostaglandin E synthase-2;
PROTAC	Proteolysis-targeting chimeras;
RBD	Receptor-binding domain;
S	Spike protein;
SARS-CoV-2	Severe Acute Respiratory Syndrome Coronavirus 2;
TEV	Tobacco etch virus;
TEVp	TEV protease;
TMPRSS2	Transmembrane protease serines 2;
TPD	Targeted protein degradation;
UPS	Ubiquitin–proteasome system;
UTRs	Untranslated regions;
VHL	von Hippel–Lindau;
vPOL	viral polymerase;
vRNA	Viral RNA;
WNV	West Nile virus;
YFV	Yellow fever virus;
ZIKV	Zika virus.
